# Supplementary material for: Randomised controlled study on the effects of pilates exercises in soccer: Comparing mat and reformer methods on physical and technical performance
Source: PLoS One. 2025 May 21;20(5):e0324129. doi: 10.1371/journal.pone.0324129 (PMC12094727; doi:10.1371/journal.pone.0324129)
Supplement: Attachment — Submitted filename: Response to Reviewers.docx [file pone.0324129.s002.docx]

**REVİEWER #1: DEAR AUTHORS:**

The following are a number of issues that prevent the manuscript from being suitable for publication. I hope you will understand that all of them are made with the sole purpose of helping you to improve your work.

**Response:** Dear reviewer, we appreciate your contributions to the our study. The suggested improvements have been implemented carefully.

Introduction

- There is a lack of contextualisation of football in the work. It is not possible to present a work on improving the performance of a sport without going into the aspects of performance in depth.

**Response:** Thank you for highlighting this inquiry. We expanded the section by clearly describing specific soccer performance metrics.

- The bibliographic selection is scarce and outdated and does not provide relevant information to contextualise the study. There are a multitude of academic studies of great impact on performance in football that are not present in the article.

**Response:** We appreciate this suggestion. We updated the references to include recent high-impact studies that clearly illustrate the connection between training methodologies and soccer performance.

- The authors point out that there is no evidence of performance improvement in football players through Pilates and focus on the differences between two Pilates methods before testing whether Pilates itself improves performance regardless of the modality.

**Response:** Thank you, we addressed how Pilates differs from other training modalities due to its unique focus on controlled core strengthening, functional stability, and movement patterns, which are particularly relevant for football players.

Material and methods

- The description of the sample indicates that the players are amateurs but does not indicate in which league they play, if they all belong to the same team, how much training load they have, and all the variables required in this type of study. To improve this point, I suggest that they be guided by published works in this respect in which all the characteristics of the participants and their conditions are indicated in detail.

**Response:** Dear Reviewer, participants are amateur soccer players competing in local amateur league. They were accustomed to a training regimen of at least six training units per week and had participated in soccer training and competitive matches for a minimum of 4 years. The experimental groups included players on the same team. The control group consisted players from a diffrent team. The research was executed out at the beginning of the season, during the preparation period. The reformer and mat pilates group were subjected to six weekly training days for eight weeks, including three Pilates and three team training days. The control group continued their regular team training regimen six days per week. Three groups were allocated one day of rest per week.

- The sample is small and by convenience, which presents a very important limitation in the results.

**Response:** Dear reviewer, we understand your worry about the sample size (N=30, split into three groups of 10 people each). For ANOVA, it is usually best to have at least 15 people in each group, but our choice is fine for a number of statistical reasons. Based on previous literature and pilot data, an effect size of 0.8 was estimated for physical performance and technical skills improvements between the intervention groups, indicating a large effect. To achieve a power of 0.80 and a significance level (alpha) of 0.05, it was calculated that a minimum of 10 participants per group would be required, totaling 30 participants across the three groups (RP, MP, and control).

- The authors state that the participants have no injuries or health problems, but do not specify how this data was obtained.

**Response:**

Yazıldı buraya eklenecek

- It is also specified that the players train regularly, but the workload is not specified.

**Response:** Dear Reviewer, players were accustomed to a training regimen of at least six training units per week and had participated in soccer training and competitive matches for a minimum of 4 years. The experimental groups included players on the same team.

The reformer and mat pilates group were subjected to six weekly training days for eight weeks, including three Pilates and three team training days. The control group continued their regular team training regimen six days per week. Three groups were allocated one day of rest per week.

- This section is disorderly and chaotic, which makes it difficult to read.

**Response:** Dear reviewer, following your comment, the methodology section of our study was revisited and further clarified.

- The exercise protocol specifies that the control group does not do any exercise while the other two groups do a protocol. This biases the results as the control group members receive less training than the groups receiving Pilates. In other words, there are two groups that do 6 sessions per week while the control group does only 3. This already generates differences, regardless of the techniques used.

**Response:** Dear Reviewer, players were accustomed to a training regimen of at least six training units per week and had participated in soccer training and competitive matches for a minimum of 4 years. The experimental groups included players on the same team.

The reformer and mat pilates group were subjected to six weekly training days for eight weeks, including three Pilates and three team training days. The control group continued their regular team training regimen six days per week. Three groups were allocated one day of rest per week.

- The authors state that the distribution of the data is normal, but do not specify the test used or add the test coefficients.

**Response:** Added (Descriptive statistical information is given in Table 1.) The distribution was within the range of (-1.5 to +1.5), indicating that the data were normally distributed [33].

**Table 3**. Descriptive statistical information

| Variables | Reformer Pilates Group | | Mat Pilates Group | | Control Group | |
| --- | --- | --- | --- | --- | --- | --- |
|  | Pre-Test  X̄ ± SD | Post-Test  X̄ ± SD | Pre-Test  X̄ ± SD | Post-Test  X̄ ± SD | Pre-Test  X̄ ± SD | Post-Test  X̄ ± SD |
| Flexibility | 33.45 ± 6.35 | 35.95± 6.08 | 30.20 ± 6.64 | 31.95 ± 6.04 | 29.55 ± 6.03 | 30.05 ± 6.04 |
| CMJ | 41.22 ± 5.59 | 45.33 ± 5.77 | 38.38 ± 6.27 | 40.72 ± 8.07 | 37.22 ± 5.97 | 37.52 ± 5.90 |
| SBJ | 212.80 ± 12.07 | 225.60 ± 12.76 | 207.90 ± 21.93 | 207.10 ± 24.88 | 207.80 ± 20.39 | 207.40 ± 19.72 |
| SLTHT R | 580.60 ± 34.88 | 620.10 ± 37.93 | 500.70 ± 65.00 | 544.70 ± 63.72 | 507.30 ± 74.43 | 516.20 ± 50.46 |
| SLTHT L | 570.20 ± 41.64 | 603.70 ± 42.43 | 507.00 ± 98.30 | 551.50 ± 76.40 | 498.80 ± 90.21 | 506.40 ± 77.13 |
| 5m sprint | 0.99 ± 0.07 | 0.95 ± 0.07 | 1.06 ± 0.73 | 0.99 ± 0.91 | 1.05 ± 0.09 | 1.03 ± 0.08 |
| 10m sprint | 1.77 ± 0.08 | 1.67 ± 0.06 | 1.78 ± 0.11 | 1.69 ± 0.09 | 1.79 ± 0.12 | 1.79 ± 0.10 |
| 20m sprint | 3.08 ± 0.10 | 2.99 ± 0.09 | 3.02 ± 0.17 | 3.00 ± 0.15 | 3.09 ± 0.17 | 3.08 ± 0.18 |
| German Agility | 7.55 ± 0.24 | 7.40 ± 0.27 | 7.91 ± 0.34 | 7.74 ± 0.29 | 7.86 ± 0.30 | 7.85 ± 0.32 |
| Balance Right Leg | 82.53 ± 8.15 | 93.18 ± 5.50 | 79.64 ± 10.06 | 89.14 ± 9.20 | 77.65 ± 10.81 | 78.21 ± 10.75 |
| Balance Left Leg | 83.32 ± 10.04 | 93.30 ± 6.84 | 80.67 ± 11.90 | 89.96 ± 7.66 | 79.28 ± 9.65 | 80.22 ± 9.24 |
| Speed Dribling | 23.47 ± 2.49 | 21.67 ± 0.73 | 24.05 ± 1.37 | 22.58 ± 0.93 | 22.05 ± 1.37 | 22.17 ± 1.34 |
| LSPT | 51.55 ± 10.71 | 39.71 ± 9.09 | 58.64 ± 12.29 | 45.90 ± 9.52 | 56.79 ± 12.17 | 57.39 ± 11.49 |
| Lobbed Passing R | 3.90 ± 1.97 | 6.60 ± 3.27 | 3.50 ± 2.46 | 5.00 ± 2.54 | 3.80 ± 1.48 | 4.10 ± 1.10 |
| Lobbed Passing L | 2.40 ± 1.96 | 2.70 ± 1.95 | 2.10 ± 2.47 | 2.20 ± 2.90 | 2.20 ± 1.62 | 1.10 ± 2.33 |
| Lobbed Pass Total | 6.30 ± 3.40 | 9.30 ± 3.77 | 5.60 ± 4.06 | 7.20 ± 2.39 | 6.00 ± 2.67 | 5.20 ± 2.04 |
| LSPT: Loughborough Soccer Passing Test, CMJ: Counter Movement Jump, SBJ: Standing Broad Jump, …SLTHT: Single Leg Triple Hop Test R: Right, L: Left, * Significant difference between groups post-training, X̄: mean, SD: Standart deviation | | | | | | |

- It is not clearly specified how the analyses have been carried out, as there is a pre-post and a comparison between groups. If all this has been done at the same time, it is more advisable to use an ANCOVA.

**Response:** Dear reviewer, we acknowledge your suggestion regarding the use of ANCOVA for pre-post comparisons alongside between-group comparisons. However, in our study, we opted for a repeated-measures two-way ANOVA (3x2) because it is an established method for analyzing repeated measures with multiple groups when assessing both within-group (pre-post) and between-group (treatment differences) effects simultaneously. ANCOVA is typically applied when there is a need to control for a continuous covariate that may influence the dependent variable. In our study, the pre-test values were already included as part of the within-subjects factor, eliminating the need for ANCOVA. Several peer-reviewed studies in similar experimental designs (small sample, pre-post intervention studies with multiple groups) have effectively used repeated-measures ANOVA without ANCOVA. Therefore, using two-way ANOVA remains a statistically sound approach in this context.

- With the sample size, I consider it much more accurate to perform non-parametric tests such as the Kruskal Wallis analysis, as ANOVA tests require a larger number of subjects. In order to use a two-way ANOVA, the groups must consist of at least 15 subjects, otherwise the results may be compromised.

**Response:** Dear reviewer, we acknowledge the concern regarding the sample size (N=30, with three groups of 10 participants each). While it is generally recommended to have at least 15 participants per group for ANOVA, several statistical considerations support our choice. First, we conducted a G*Power analysis, which confirmed that our sample size was adequate to detect meaningful effects with sufficient statistical power. Second, before applying parametric tests, we rigorously assessed normality, skewness, and kurtosis to ensure our data met ANOVA assumptions. Our results fell within the acceptable range (-1.5 to +1.5), supporting the validity of using ANOVA. Third, many published articles with similar sample sizes (N = 30) have used parametric tests, including ANOVA, demonstrating its acceptability in experimental settings. Finally, non-parametric tests, such as the Kruskal-Wallis test, do not account for within-subject dependencies (pre-post measures), making them less suitable for repeated-measures designs. Considering these factors, ANOVA remains a valid choice for our analysis.

***References for this section:***

1. Field, A. (2018). Discovering Statistics Using IBM SPSS Statistics. Sage Publications.

2. Tabachnick, B. G., Fidell, L. S., & Ullman, J. B. (2019). Using multivariate statistics (Vol. 6, pp. 497-516).

3. Maxwell, S. E., Delaney, H. D., & Kelley, K. (2017). Designing experiments and analyzing data: A model comparison perspective. Routledge.

4. Faul, F., Erdfelder, E., Lang, A. G., & Buchner, A. (2007). G* Power 3: A flexible statistical power analysis program for the social, behavioral, and biomedical sciences. Behavior research methods, 39(2), 175-191.

5. Johnson, R. A., & Wichern, D. W. (2002). Applied multivariate statistical analysis.

- The results are confusing and disorganised

**Response:** Thank you for this recommendation. We have separated the conclusions from the discussion, creating a distinct Conclusions section at the end of the manuscript to enhance clarity and structure.

I hope that these indications will help you to improve your work.

Kind regards

**REVİEWER #2: OVERVİEW:**

The article presents a randomized controlled trial comparing the effects of reformer pilates (RP) and mat pilates (MP) exercises on the physical performance and technical skills of soccer players. The study reveals that both pilates methods improve the performance of soccer players, but reformer pilates is more effective than mat pilates. The article provides valuable information, especially for those working in the field of sports science and training methodology.

Thank you for the interesting insights your article provides. The manuscript is well-written and deals with an important topic. I have thoroughly reviewed the manuscript and would like to make some suggestions for your review.

**Response:** Dear reviewer, thank you very much for your valuable opinions and contributions to the article. The revisions you indicated have been made.

Abstract section:

- RP group (n=10; 20.60 ± 1.65), MP group (n=10; 19.40 ± 1.35), and control group (CG) (n=10; 20.10 ± 1.15).

- Add unit, example: (age 20.60 ± 1.65 years)

**Response:** Added

Thirty voluntary participants were randomly assigned to either RP group (n=10; age = 20.60 ± 1.65), MP group (n=10; age = 19.40 ± 1.35), and control group (CG) (n=10; age = 20.10 ± 1.15).

Introduction section:

- “Soccer players must have a high degree of athletic performance characteristics such as including balance, coordination, flexibility, agility, speed, endurance, and technical skills in order to properly accomplish these activities (Arslan et al., 2021). Pilates exercises are used as a training method to improve these features (Yilmaz et al., 2023; Nogueira et al., 2022; Chinnavan et al., 2015).

add a reference to this sentence.

**Response:** Dear reviewer this references added

- add a hypothesis sentence

**Response:** We appreciate this suggestion. A clear hypothesis will be included to clarify the expected outcomes based on existing evidence.

method:

Learn more about the study design. When and in what order were the tests performed? Indicate what time of the day the training and tests were performed.

Learn more about soccer training

**Response:** Dear reviewer study design added.

Learn more about the “German Agility” test protocol.

**Response:** Dear reviewer this section is revised

This agility test is one of the tests used to determine motor diagnostics in the German football talent identification and development program. The participant begins the race from a stationary position, without any cues, and navigates through a slalom course to reach the finish line as quickly as possible. It is utilized on a track measuring 13 meters in length and 4 meters in wide. Slalom bars are positioned at the 3rd, 4th, and 5th meters along the 4th wide meter and a long with at the 0th, 8th, 9th, and 10th meters. The slalom bars at the 4th and 9th meters are positioned 0.50 cm inward You can access the test visual in the validity and reliability article (Höner et al., 2015).

**REVİEWER #3:**

Thank you for the opportunity to review the manuscript entitled - Randomised Controlled Study on the Effects of Pilates Exercises in Soccer: Comparing Mat and Reformer Methods on Physical and Technical Performance

The study is interesting and addresses a topical scientific topic.

**Response:** Dear reviewer, thank you very much for your valuable opinions and contributions to the article. The revisions you indicated have been made.

Recommendations for improving the content of the manuscript:

Introduction:

• To detail the connection between pilates and soccer with a focus on the physical and technical components of the study. To argue why pilates was chosen and not other aerobic gymnastics or stretching programs.

**Response:** Thank you for the above comment, we revised it accordingly with a focus on the physical and technical components of the study

• To detail more specifically the novel aspects of the present study in relation to previous studies on the same topic.

**Response:** We clarified that the current study uniquely compares two distinct Pilates modalities (mat vs. reformer) directly within the same experimental design in the context of soccer performance.

Materials and Methods:

• Participant and Randomisation – this section should be restructured, as it contains repeated details.

**Response:** Dear reviewer, this section is revised. Participant and randomization sections were combined and duplicate sections were removed.

• Study design – to add this subsection where the periodization of the study will be detailed: year, testing periods, and stages of the study (part of the content of this section is also found in the Introduction and Randomization)

**Response:** Dear reviewer, study design section was added.

Result – this section is well structured and interpreted.

Response: Dear reviewer, thank you so much your good comments.

Discussions:

- To add at the end of the Discussions - the practical implications and limits of the study based on the relevant results.

**Response:** We appreciate this suggestion and have revised the manuscript by clearly adding practical implications and limitations at the end of the discussion section, as recommended.

Conclusions

- They recommended separating the text from the Discussions and including the Conclusions section, as well as the separate section at the end of the study.

**Response:** Thank you for this recommendation. We have separated the conclusions from the discussion, creating a distinct Conclusions section at the end of the manuscript to enhance clarity and structure.
